# Experimental observation of the *ν*_1_+3*ν*_3_ combination bands of ^16^O^14^N^18^O and ^18^O^14^N^18^O in the near infrared spectral region

**DOI:** 10.1016/j.heliyon.2024.e24853

**Published:** 2024-01-24

**Authors:** S. Chandran, J. Orphal, A.A. Ruth

**Affiliations:** a School of Physics & Environmental Research Institute, University College Cork, Cork, Ireland; b Division 4 “Natural and Built Environment”, Karlsruhe Institute of Technology (KIT), Kaiserstrasse 12, 76131, Karlsruhe, Germany

**Keywords:** Cavity enhanced absorption spectroscopy, Fourier transform (FT), FT-IBBCEAS, Near infrared (IR), Nitrogen dioxide, Isotopologues, Combination bands

## Abstract

The first observation of the *ν*_1_+3*ν*_3_ combination band of the nitrogen dioxide isotopologue ^16^O^14^N^18^O is presented. The band was measured using Fourier-Transform Incoherent Broad-Band Cavity Enhanced Absorption Spectroscopy (FT-IBBCEAS) in the region between 5870 cm^−1^ and 5940 cm^−1^. To confirm the assignment, the band was simulated using a standard asymmetric top Watson Hamiltonian using extrapolated rotational and centrifugal distortion constants. Furthermore, the first experimental observation of the *ν*_1_+3*ν*_3_ band of the ^18^O^14^N^18^O isotopologue is also reported. The positions of ro-vibrational lines of the *ν*_1_+3*ν*_3_ band of the naturally most abundant isotopologue ^16^O^14^N^16^O were used for wavenumber calibration of line positions.

## Highlights

• New bands of NO_2_ isotopologues in the near IR measured using FT-incoherent broadband cavity enhanced absorption spectroscopy.
• First experimental observation of the *ν*_1_+3*ν*_3_ band of ^16^O^14^N^18^O.
• Center position of the *ν*_1_+3*ν*_3_ band of ^18^O^14^N^18^O confirmed.
• Simulation with standard asymmetric top Watson Hamiltonian confirms assignment of ^16^O^14^N^18^O band.

## Graphical abstract

**Figure:**
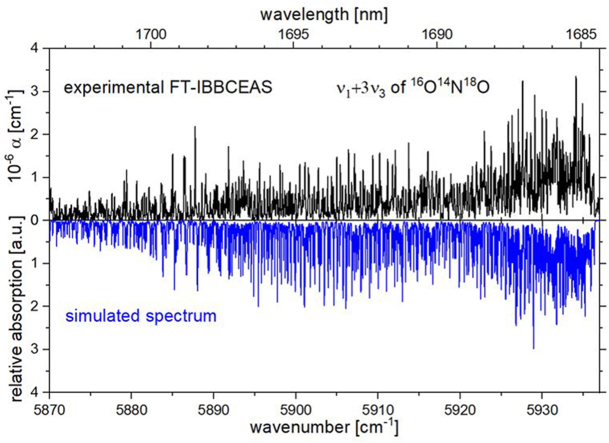

## 1 Introduction

Nitrogen dioxide (NO_2_) has been extensively studied spectroscopically owing to its importance as atmospheric trace gas constituent and as driver of a large number of atmospheric gas phase reactions [[1], [2], [3]]. The detection of NO_2_ in the atmosphere is commonly based on its strong absorption spectrum in the blue region of the visible spectrum between ∼400 and 450 nm [[4], [5], [6]]. In addition, the mid-infrared absorption bands of NO_2_ at 3.4 and 6.2 μm have also been used [7,8]. Apart from its atmospheric importance NO_2_ is also interesting from a molecular spectroscopy point of view and therefore has been extensively studied by high-resolution laboratory spectroscopy in the infrared region [[9], [10], [11], [12], [13], [14], [15], [16], [17], [18], [19], [20], [21], [22], [23], [24], [25], [26], [27], [28], [29], [30], [31], [32], [33], [34], [35]]. Based on the substantial amount of experimental data on NO_2_, Lukashevskaya et al. [30] established an extensive list of ro-vibrational line positions and intensities of N^16^O_2_ from the far- to the near-IR (0.006–7916 cm^−1^). NO_2_ lines are also included in the HITRAN [36] and GEISA spectroscopic databases [37,38]. In the near-infrared spectral region the strongest band of NO_2_ is the *ν*_1_+3*ν*_3_ combination band, which was first identified in 1958 by Arakawa and Nielsen [9]. Olman and Hause also observed this band again in 1968 [10] and Blank et al. carried out a first detailed rotational analysis of this band in 1970 [11]. More recently, Miljanic et al. [24] presented a very detailed experimental and theoretical study of the *ν*_1_+3*ν*_3_ band of N^16^O_2_; in Ref. [24] 1147 ro-vibrational transitions were identified with rotational quantum numbers N and K_a_ of up to 47 and 8, respectively. Several years later, Naumenko et al. [39] published also a study of this band and reported experimental line positions and intensities of 3154 transitions with quantum numbers N and K_a_ of up to 59 and 13, respectively.

The near infrared region is spectroscopically particularly interesting. Around only 10000 cm^−1^, the two lowest electronic states of NO_2_ cross with a conical intersection [21,40]. This conical intersection leads to unusual effects including a breakdown of standard spectroscopic theory [21], so that the line-by-line analysis of the rovibronic bands of the A-X transition in the near-infrared (although fully resolved at Doppler-limited resolution [21]) has not been successful until today. A proper understanding of the vibrational structure of the electronic ground state of NO_2_ is therefore very important. For this, experimental observations of isotopically substituted species of NO_2_ at high energies are most suitable. However, despite the extensive spectroscopic literature on the most abundant isotopologue N^16^O_2_, spectroscopic investigation of other isotopologues, especially those with singly and doubly substituted oxygen (^16^O^14^N^18^O and ^18^O^14^N^18^O) are sparse [41]. In 1976, Hardwick and Brand [42] predicted the band centers of 25 bands of ^18^O^14^N^18^O between 722 cm^−1^ and 5854 cm^−1^. In 2006, Volkers et al. measured the high-resolution spectra of N^16^O_2_, ^16^O^14^N^18^O, ^18^O^14^N^18^O in the spectral region between 11800 and 14380 cm^−1^ [40]. More recently Marinina et al. [43] reported the Fourier transform IR spectra in the 1540–1640 cm^−1^ spectral region were the *ν*_3_ band of ^16^O^14^N^18^O is located.

In this work, we report the first experimental observation of the *ν*_1_+3*ν*_3_ bands of the isotopologues ^16^O^14^N^18^O and ^18^O^14^N^18^O in the near infrared region between 5840 cm^−1^ and 6000 cm^−1^ using Fourier Transform-Incoherent Broad-Band Cavity-Enhanced Absorption Spectroscopy (FT-IBBCEAS) [44,45]. In comparison to the well-known *ν*_1_+3*ν*_3_ band center of ^14^N^16^O_2_ at 5984.7 cm^−1^ [24,39], the corresponding band centers of ^16^O^14^N^18^O and ^14^N^18^O_2_ were observed at 5922 cm^−1^ and 5854 cm^−1^, respectively.

## 2 Experimental

### 2.1 Measurement method, components, and parameters

The general experimental setup has been published before [[46], [47], [48]] and thus merely the key experimental aspects are outlined here. The light source was a 2 W supercontinuum source (Fianium SC 450-2) operating at a repetition rate of 80 MHz. The broadband light (500–1800 nm) was passed through a long pass filter (Thorlabs FEL 1250-1) with a cut-off wavelength of 1250 nm (8000 cm^−1^). The light was spatially filtered and collimated before entering the optical cavity of length *d* ∼644 cm. The optical cavity consisted of two dielectric plano-concave mirrors (Layertec GmbH, Germany) with a reflectivity of ∼0.999 between 5750 cm^−1^ and 8000 cm^−1^. The optical cavity was attached to a vacuum chamber consisting of long stainless-steel pipes, which was evacuated by a turbo pump (Leybold Turbovac) to a pressure of approximately 10^−5^ mbar before injecting any gas samples. The experiment was carried out with a static gas and hence the cavity mirrors were not purged. The light exiting the cavity was coupled into a multimode fiber with an IR optimized achromatic doublet. The other end of the multimode fiber was connected to the entrance port (aperture diameter 0.5 mm) of a Fourier transform spectrometer (FTS; Bruker Vertex 80). The light transmitted by the cavity was measured without (evacuated cavity ∼10^−5^ mbar), *I*_0_(*λ*), and with the sample, *I*(*λ*), in the cavity. From the ratio of the transmission intensities, the reflectivity of the mirrors *R*(*λ*), and the sample path length per pass, *d*, inside the optical cavity, the absorption coefficient of the sample *α*(*λ*) was evaluated using [49]:

(1) $$\alpha \left(\lambda \right)=\left(\frac{I_{0}\left(\lambda \right)}{I\left(\lambda \right)}-1\right)\frac{\left(1-R\left(\lambda \right)\right)}{d}\text{.}$$

The instrumental line shape and spectral resolution of 0.08 cm^−1^ was obtained from the measurement of a CO_2_ spectrum at 10.7 mbar employing Norton-Beer weak apodization. The integration time used for measuring the spectrum was 120 min. For this integration time a signal-to-noise-ratio of >198 was achieved, which was evaluated on the basis of the ^16^O^14^N^18^O absorption at ∼5934 cm^−1^.

### 2.2 Materials and sample preparation

CO_2_ (purity >99.90 %) was purchased from Irish Oxygen and used without further purification. H_2_^18^O and NO_2_ were purchased from Taiyo Nippon Sanso Corporation (purity >98 %) and Sigma Aldrich (purity >99.994 %), respectively. The H_2_^18^O samples were degassed by several “freeze-pump-thaw” cycles before injection into the cavity. The cavity chamber was first evacuated to a pressure of ∼10^−5^ mbar at room temperature. It was then primed with H_2_^18^O vapor at a pressure of ∼6.8 mbar. A waiting time of 1 h was provided for H_2_^18^O to thermally equilibrate and passivate the chamber walls. After 1 h the pressure dropped to ∼6.2 mbar. Subsequently NO_2_ was injected (partial pressure ∼3.5 mbar) into the chamber. The mixture was left to equilibrate for approximately 60 min to enable the ^18^O exchange and formation of the NO_2_ isotopologues ^16^O^14^N^18^O and ^18^O^14^N^18^O at room temperature through the formation and decomposition of ^18^O-substituted HONO and HNO_3_. The overall pressure of the mixture at the start of the measurement reduced to 8.2 mbar.

### 2.3 Reflectivity calibration

To retrieve absolute absorption coefficients with FT-IBBCEAS, the broadband mirror reflectivity must be established [45]. The mirror reflectivity, *R*(*λ*), was calibrated by filling a well evacuated (∼10^−5^ mbar) cavity with a known amount of CO_2_ (∼10.7 mbar) [44,46]. Based on Eq. (1), the reflectivity was determined using the measured CO_2_ absorption coefficients and the literature absorption cross-section of CO_2_ from the HITRAN database [36]. *R*(*λ*) was found to be almost constant in the wavenumber region from 5840 to 6000 cm^−1^ with a maximum value of 0.9982 falling off by ca. 0.0005 at the edges of the region. The uncertainty in the reflectivity calibration (based on errors in cross-sections and pressure measurement of CO_2_) leads to an uncertainty in (1-*R*) of ∼10 %. This uncertainty is the dominant systematic error contribution to the measured NO_2_ absorption coefficients. Other uncertainties occur from the pressure measurements (∼5 %) and from the average intensity fluctuation of the supercontinuum light source (∼4 %) [28]. The total Gaussian absolute uncertainty contributing to the measured absorption coefficients was estimated to be ∼12 %.

## 3 Results and discussion

**Fig. 1:**
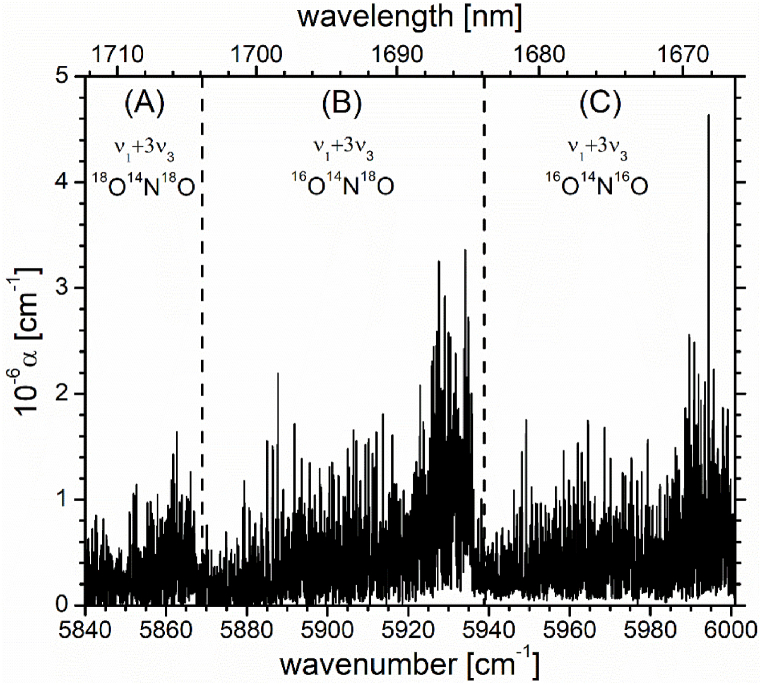
Overview of the FT-IBBCEA spectrum in the region between 5840 cm^−1^ and 6000 cm^−1^ measured with a resolution of 0.08 cm^−1^. Sections (A), (B), and (C), separated by vertical dashed lines, represent the *ν*_1_+3*ν*_3_ combination overtone bands of ^18^O^14^N^18^O, ^16^O^14^N^18^O, and ^16^O^14^N^16^O, respectively. The measurement time was 120 min.

Fig. 1 shows the experimental FT-IBBCEA spectrum of the absorption coefficient of NO_2_ at 8.2 mbar (total absolute pressure) in the region of the *ν*_1_+3*ν*_3_ band between 5840 cm^−1^ and 6000 cm^−1^. The spectrum clearly exhibits three spectrally overlapping bands that are attributed to the isotopologues ^18^O^14^N^18^O, ^16^O^14^N^18^O, and ^14^N^16^O_2_. The spectrum will be further discussed after a short description of aspects concerning the wavenumber calibration; the raw data are also available as supplementary material.

### 3.1 Line position accuracy

Wavenumber calibration is a crucial aspect in high resolution spectroscopy. As a first step, to minimize the error in line position, the wavenumbers of 20 strong ro-vibrational absorption lines of the 3*ν*_1_ band of CO_2_ within the reflectivity calibration spectrum between 6040.7 and 6106.7 cm^−1^ were compared with the corresponding line positions reported in the HITRAN data base [36]. The band center of the 3*ν*_1_ band of CO_2_ is about 150 cm^−1^ above the band center of the new ^16^O^14^N^18^O band reported in this work. The observed average absolute discrepancy between the measured and the HITRAN line positions was 0.019 ± 0.003 cm^−1^. This discrepancy is about 4 times smaller than the instrumental resolution of 0.08 cm^−1^, which is indeed satisfying given the signal-to-noise-ratio and rather high line density in the experimental spectrum.

**Fig. 2:**
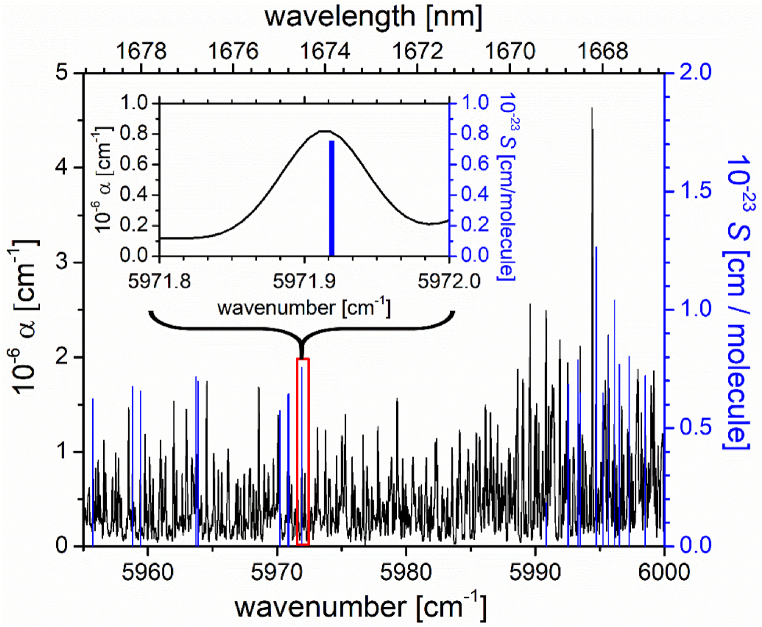
Comparison of 20 line positions in the *ν*_1_+3*ν*_3_ band of N^16^O_2_ between 5955 cm^−1^ and 6000 cm^−1^ from section (C) of Fig. 1 (black trace) with the corresponding ro-vibrational features of the same band reported in Naumenko et al. 2019 [39] (blue trace, cut off intensity >1.8 × 10^−24^ cm/molecule). The inset shows a magnified view of a line at 5971.92 cm^−1^ (red rectangle) illustrating a typical match of position.

Based on the wavenumber calibration of the Fourier transform spectrum using the CO_2_ spectrum, the wavenumber scale accuracy was independently verified by comparing 20 strong ro-vibrational absorption features of the *ν*_1_+3*ν*_3_ band of N^16^O_2_ reported in Ref. [37] with the experimental lines measured in the present study. Fig. 2 shows the selected lines in the *ν*_1_+3*ν*_3_ band of N^16^O_2_ reported by Naumenko et al. 2019 [39] (cut off intensity >1.8 × 10^−24^ cm/molecule) together with the FT-IBBCEAS spectrum in the region between 5955 cm^−1^ and 6000 cm^−1^ (see section (C) in Fig. 1). Note that, the cavity ring-down spectrum in Ref. [39] has a five times higher resolution (0.015 cm^−1^) than our FT-IBBCEA spectrum (0.08 cm^−1^). Consequently, in regions with very high line density, ro-vibrational features in the CRD spectrum generally overlap with the ro-vibrational absorption features of the FT-IBBCEA spectrum. The 20 lines were carefully selected in wavenumber regions where the spectrum is less congested. A typical example illustrating the match of the center wavelength of our data with those from Ref. [39] (stick spectrum representing line intensity) is shown in the inset of Fig. 2 (red rectangle magnified).

Table 1 contains the positions from this study (ν_exp_) and from Naumenko et al. (ν_N_) [39], the position differences (Δν) and the rotational assignments (*N*, *Ka*, *Kc*) in the lower and upper state of the 20 selected ro-vibrational lines. The average absolute discrepancy between the measured and the literature line positions of N^16^O_2_ was 0.018 ± 0.009 cm^−1^. The *ν*_1_+3*ν*_3_ band of N^16^O_2_, used for wavenumber scale calibration, is adjacent (∼60 cm^−1^) to the *ν*_1_+3*ν*_3_ band of the ^16^O^14^N^18^O isotopologue. Therefore, the wavenumber calibration is expected to also hold in the region of the corresponding ^16^O^14^N^18^O band assuming a calibration uncertainty of the new *ν*_1_+3*ν*_3_ lines of ^16^O^14^N^18^O to be about 0.027 cm^−1^ (=max error for N^16^O_2_ from the literature). Note that this uncertainty is again small in comparison with the spectral resolution of 0.08 cm^−1^, and in very good agreement with the value determined from the spectral calibration using CO_2_.

**Table 1:** Comparison of 20 ro-vibrational line positions of the (1 0 3) ← (0 0 0) band of N^16^O_2_ measured using FT-IBBCEAS (column 1) and CRDS from Ref. [39] (column 2). The differences between the experimental line positions in this work and the line positions from the literature are shown in column 3. Rotational assignments are given in columns 4 and 5.

FT-IBBCEAS this work	CRDS Naumenko et al. 2019 [39]	Difference	Rotational quantum numbers [39]	
ν_exp_ [cm^−1^]	ν_N_ [cm^−1^]	Δν = ν_N_ − ν_exp_ [cm^−1^]	Upper (*N, Ka, Kc)*	Lower (*N, Ka, Kc)*
5955.759	5955.76940	0.010	(25 0 25)	(26 0 26)
5958.818	5958.83280	0.015	(21 2 19)	(22 2 20)
5959.428	5959.44200	0.014	(22 1 22)	(23 1 23)
5963.737	5963.74740	0.011	(19 0 19)	(20 0 20)
5963.865	5963.88720	0.022	(17 2 15)	(18 2 16)
5970.200	5970.21670	0.016	(13 1 12)	(14 1 13)
5970.810	5970.83070	0.020	(11 2 9)	(12 2 10)
5970.923	5970.93680	0.013	(13 0 13)	(14 0 14)
5971.910	5971.91880	0.009	(10 2 9)	(11 2 10)
5990.833	5990.86850	0.035	(32 4 29)	(31 4 28)
5992.506	5992.54290	0.037	(11 0 11)	(10 0 10)
5993.312	5993.30770	−0.004	(13 1 12)	(12 1 11)
5993.470	5993.46090	−0.009	(14 1 14)	(13 1 13)
5994.705	5994.72140	0.016	(15 0 15)	(14 0 14)
5995.225	5995.25620	0.031	(21 2 19)	(20 2 18)
5995.647	5995.67160	0.025	(17 0 17)	(16 0 16)
5996.114	5996.13550	0.021	(20 1 20)	(19 1 19)
5996.498	5996.52580	0.028	(19 0 19)	(18 0 18)
5997.274	5997.27880	0.005	(21 0 21)	(20 0 20)
5998.502	5998.51020	0.008	(25 0 25)	(24 0 24)

### 3.2 The *ν*_1_+3*ν*_3_ band of ^18^O^14^N^18^O

Section (A) of Fig. 1 shows the *ν*_1_+3*ν*_3_ band of ^18^O^14^N^18^O in the region between 5840.0 cm^−1^ and 5868.0 cm^−1^. In 1976, using an anharmonic force field calculated from experimental spectra, Hardwick and Brand [42] predicted the center of the *ν*_1_+3*ν*_3_ band of ^18^O^14^N^18^O (see Tab. 3 in Ref. [42]) to be at 5853 cm^−1^, which is indeed in excellent agreement with our observation (see the spectrum in Fig. 1) at **∼**5854 cm^−1^. Our measurement is hence an experimental corroboration of a prediction made almost 50 years ago. The results in Ref. [42] were based on an anharmonic potential calculation from experimental data of the N^16^O_2_ isotopologue.

### 3.3 The *ν*_1_+3*ν*_3_ band of ^16^O^14^N^18^O

**Fig. 3:**
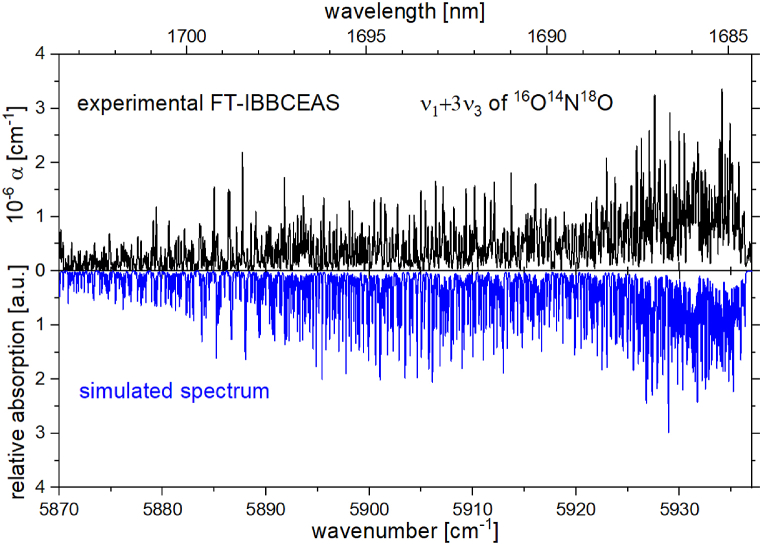
The upper panel shows the *ν*_1_+3*ν*_3_ band of ^16^O^14^N^18^O (also see section (B) of Fig. 1) measured using FT-IBBCEAS. The lower panel (blue trace) shows the simulated spectrum of the same band.

Section (B) of Fig. 1 shows the *ν*_1_+3*ν*_3_ band of ^16^O^14^N^18^O in the region between 5869.8 cm^−1^ and 5936.3 cm^−1^. To confirm this assignment, Fig. 3 presents the experimentally observed *ν*_1_+3*ν*_3_ band of ^16^O^14^N^18^O (upper panel, black trace), and a simulated spectrum of this band (lower panel, blue trace). The simulation was made using an A-reduced Watson-type Hamiltonian, using the values of Bird et al. [50] for the principal rotational constants, and of Miljanic et al. [24] for the centrifugal distortion constants, of the ground state. Based on the vibrational dependence of these constants from Miljanic et al. [24] the rotational constants of the excited (1 0 3) state (see Table 2), were calculated. The intensities were calculated using the rigid-rotor approximation based on the symmetric top wavefunctions obtained from the line position calculation. Although the simulation is not based on a line-by-line fit of the observed spectrum, the overall agreement is satisfying, since it clearly confirms the assignment to the *ν*_1_+3*ν*_3_ band of ^16^O^14^N^18^O. By comparing the observed spectrum with the simulation, the band center was identified to be located at 5922.3 cm^−1^. A list of line positions is also given in Table S1 in the supplementary material. A line-by-line analysis of this combination band was not possible since the spectral resolution of our data is limited and the electron spin splitting constants for ^16^O^14^N^18^O are not known for the lower state.

**Table 2:** Band centers, rotational [50] and centrifugal distortion constants [24] of the ground (0 0 0) and excited (1 0 3) states of ^16^O^14^N^18^O used for calculating the simulated spectrum in Fig. 3.

Spectroscopic constants	Ground state (0 0 0)	Excited state (1 0 3)
	[cm^−1^]	[cm^−1^]
A	7.878664955	7.287696365
B	0.409797162	0.399355884
C	0.388673677	0.377484857
D_J_	2.992447 × 10^−7^	3.165780 × 10^−7^
D_JK_	−1.968220 × 10^−5^	−2.675680 × 10^−5^
D_K_	2.687876 × 10^−3^	2.391340 × 10^−3^
d_J_	3.192774 × 10^−8^	3.881700 × 10^−8^
d_K_	4.054700 × 10^−6^	5.509000 × 10^−6^
*ν*_0_	0	5922.3

## 4 Conclusions

In this publication, the first experimental observation of the *ν*_1_+3*ν*_3_ combination bands of ^16^O^14^N^18^O (5922 cm^−1^) and ^18^O^14^N^18^O (5854 cm^−1^) in the near infrared is reported using FT-IBBCEAS. The band center for the doubly substituted species confirms the prediction of Hardwick and Brand [42] made nearly 50 years ago. The study demonstrates again the potential of FT-IBBCEAS for studying weak absorption bands in the near-infrared at high spectral resolution and at large spectral bandwith.

## Data availability statement

This article presents the data generated and analyzed in three figures and two tables. The data of the spectrum in Fig. 1 are provided in the supplementary material together with a line list of ro-vibrational lines of the *ν*_1_+3*ν*_3_ combination band of ^16^O^14^N^18^O. Additional information on methods or materials used in this study will be made available upon request to the corresponding author.

## CRediT authorship contribution statement

**S. Chandran:** Writing – original draft, Methodology, Investigation, Formal analysis, Data curation. **J. Orphal:** Writing – review & editing, Software, Formal analysis, Conceptualization. **A.A. Ruth:** Writing – original draft, Visualization, Supervision, Resources, Project administration, Methodology, Funding acquisition, Conceptualization.

## Declaration of competing interest

The authors declare that they have no known competing financial interests or personal relationships that could have appeared to influence the work reported in this paper.

## References

[1] Crutzen P.J. (1970). The influence of nitrogen oxides on the atmospheric ozone content. Q. J. R. Meteorol. Soc..

[2] Dunlea E.J., Herndon S.C., Nelson D.D., Volkamer R.M., San Martini F., Sheehy P.M., Zahniser M.S., Shorter J.H., Wormhoudt J.C., Lamb B.K., Allwine E.J., Gaffney J.S., Marley N.A., Grutter M., Marquez C., Blanco S., Cardenas B., Retama A., Ramos Villegas C.R., Kolb C.E., Molina L.T., Molina M.J. (2007). Evaluation of nitrogen dioxide chemiluminescence monitors in a polluted urban environment. Atmos. Chem. Phys..

[3] Kley D., McFarland M. (1980). Chemiluminescence detector for NO and NO_2_. Atmos. Tech..

[4] Fuchs H., Dubé W.P., Lerner B.M., Wagner N.L., Williams E.J., Brown S.S. (2009). A sensitive and versatile detector for atmospheric NO_2_ and NO_X_ based on blue diode laser cavity ring-down spectroscopy. Environ. Sci. Technol..

[5] Chandran S., Puthukkudy A., Varma R. (2017). Dual-wavelength dual-cavity spectrometer for NO_2_ detection in the presence of aerosol interference. Appl. Phys. B.

[6] Chen J., Wang D.-N., Ramachandran A., Chandran S., Li M., Varma R. (2020). An open-path dual-beam laser spectrometer for path-integrated urban NO_2_ sensing. Sens. Actuators A: Phys..

[7] López-Puertas M., Funke B., Clarmann T.V., Fischer H., Stiller G.P. (2006). The stratospheric and mesospheric NOy in the 2002–2004 polar winters as measured by MIPAS/ENVISAT. Space Sci. Rev..

[8] Flaud J.M., Camy‐Peyret C., Brault J.W., Rinsland C.P., Cariolle D. (1988). Nighttime and daytime variation of atmospheric NO_2_ from ground‐based infrared measurements. Geophys. Res. Lett..

[9] Arakawa E.T., Nielsen A.H. (1958). Infrared spectra and molecular constants of ^14^NO_2_ and ^15^NO_2_. J. Mol. Spectrosc..

[10] Olman M.D., Hause C.D. (1968). Molecular constants of nitrogen dioxide from the near infrared spectrum. J. Mol. Spectrosc..

[11] Blank R.E., Olman M.D., Hause C.D. (1970). Upper state molecular constants for the (0, 0, 3) and (1, 0, 3) vibration-rotation bands of nitrogen dioxide. J. Mol. Spectrosc..

[12] Perrin A., Flaud J.M., Camy-Peyret C., Carli B., Carlotti M. (1988). The far infrared spectrum of ^14^N^16^O_2_. Mol. Phys..

[13] Perrin A., Camy-Peyret C., Flaud J.M., Kauppinen J. (1988). The ν_2_ band of ^14^N^16^O_2_ - spin-rotation perturbations in the (010) state. J. Mol. Spectrosc..

[14] Semmoud-Monnanteuil N., Colmont J.M., Perrin A., Flaud J.M., Camy-Peyret C. (1989). New measurements in the millimeter-wave spectrum of ^14^N^16^O_2_. J. Mol. Spectrosc..

[15] Delon A., Jost R. (1991). Laser induced dispersed fluorescence spectra of jet cooled NO_2_: the complete set of vibrational levels up to 10000 cm^-1^ and the onset of the ${\tilde{X}}^{2}A_{1}\;-\;{\tilde{A}}^{2}B_{2}$ vibronic interaction. J. Chem. Phys..

[16] Perrin A., Flaud J.M., Camy-Peyret C., Vasserot A.M., Guelachvili G., Goldman A., Murcray F.J., Blatherwick R.D. (1992). The ν_1_, 2ν_2_, and ν_3_ interacting bands of ^14^N^16^O_2_: line positions and intensities. J. Mol. Spectrosc..

[17] Perrin A., Flaud J.M., Camypeyret C., Goldman A., Murcray F.J., Blatherwick R.D., Rinsland C.P. (1993). The ν_2_ and 2ν_2_-ν_2_ bands of ^14^N^16^O_2_: electron spin-rotation and hyperfine contact resonances in the (010) vibrational state. J. Mol. Spectrosc..

[18] Perrin A., Flaud J.M., Camypeyret C., Hurtmans D., Herman M., Guelachvili G. (1994). The ν_2_ +ν_3_ and ν_2_+ν_3_-ν_2_ bands of ^14^N^16^O_2_: line positions and intensities. J. Mol. Spectrosc..

[19] Perrin A., Flaud J.M., Camy-Peyret C., Hurtmans D., Herman M. (1996). The {2ν_3_, 4ν_2_, 2ν_2_+ ν_3_} and 2ν_3_-ν_3_ bands of ^14^N^16^O_2_: line positions and intensities. J. Mol. Spectrosc..

[20] Mandin J.Y., Dana V., Perrin A., Flaud J.M., Camy-Peyret C., Régalia L., Barbe A. (1997). The {ν_1_+ 2ν_2_, ν_1_+ ν_3_} bands of ^14^N^16^O_2_: line positions and intensities; Line intensities in the ν_1_+ν_2_+ν_3_-ν_2_ hot band. J. Mol. Spectrosc..

[21] Orphal J., Dreher S., Voigt S., Burrows J., Jost R., Delon A. (1998). The near-infrared bands of NO_2_ observed by high-resolution Fourier-transform spectroscopy. J. Chem. Phys..

[22] Liu Y., Liu X., Liu H., Guo Y. (2000). A global analysis of pure rotational spectra of ^14^N^16^O_2_ in the ground vibrational state. J. Mol. Spectrosc..

[23] Stephen T.M., Goldman A., Perrin A., Flaud J.M., Keller F., Rinsland C.P. (2000). New high-resolution analysis of the 3ν_3_ and 2ν_1_+ν_3_ bands of nitrogen dioxide (NO_2_) by Fourier transform spectroscopy. J. Mol. Spectrosc..

[24] Miljanic S., Perrin A., Orphal J., Fellows C.E., Chelin P. (2008). New high-resolution analysis of the ν_1_+3ν_3_ band of nitrogen dioxide in the near infrared spectral region. J. Mol. Spectrosc..

[25] Perrin A., Kassi S., Campargue A. (2010). First high-resolution analysis of the 4ν_1_+ν_3_ band of nitrogen dioxide near 1.5 μm. J. Quant. Spectrosc. Radiat. Transf..

[26] Mondelain D., Perrin A., Kassi S., Campargue A. (2012). First high-resolution analysis of the 5ν_3_ band of nitrogen dioxide near 1.3 μm. J. Quant. Spectrosc. Radiat. Transf..

[27] Lukashevskaya A.A., Naumenko O.V., Perrin A., Mondelain D., Kassi S., Campargue A. (2013). High sensitivity cavity ring down spectroscopy of NO_2_ between 7760 and 7917 cm^-1^. J. Quant. Spectrosc. Radiat. Transf..

[28] Raghunandan R., Perrin A., Ruth A.A., Orphal J. (2014). First analysis of the 2ν_1_+3ν_3_ band of NO_2_ at 7192.159 cm^-1^. J. Mol. Spectrosc..

[29] Gueye F., Kwabia Tchana F., Landsheere X., Perrin A. (2014). New line positions analysis of the ν_1_+ν_2_+ν_3_ band of NO_2_ at 3637.848cm^-1^. J. Quant. Spectrosc. Radiat. Transf..

[30] Lukashevskaya A.A., Lyulin O.M., Perrin A., Perevalov V.I. (2015). Global modeling of NO_2_ line positions. Atmos. Ocean. Opt..

[31] Lukashevskaya A.A., Naumenko O.V., Mondelain D., Kassi S., Campargue A. (2016). High sensitivity cavity ring down spectroscopy of the 3ν_1_+3ν_2_+ν_3_ band of NO_2_ near 7587 cm^-1^. J. Quant. Spectrosc. Radiat. Transf..

[32] Lukashevskaya A.A., Naumenko O.V., Kassi S., Campargue A. (2017). First detection and analysis of the 3ν_1_+ν_2_+ν_3_ band of NO_2_ by CRDS near 6156 cm^-1^. J. Mol. Spectrosc..

[33] Lukashevskaya A.A., Kassi S., Campargue A., Perevalov V.I. (2017). High sensitivity cavity ring down spectroscopy of the 2ν_1_ +3ν_2_ +ν_3_ band of NO_2_ near 1.57 μm. J. Quant. Spectrosc. Radiat. Transf..

[34] Lukashevskaya A.A., Kassi S., Campargue A., Perevalov V.I. (2017). High sensitivity cavity ring down spectroscopy of the 4ν_3_ band of NO_2_ near 1.59 μm. J. Quant. Spectrosc. Radiat. Transf..

[35] Lukashevskaya A.A., Mondelain D., Campargue A., Perevalov V.I. (2018). High sensitivity cavity ring down spectroscopy of the ν_1_+4ν_3_ band of NO_2_ near 1.34 μm. J. Quant. Spectrosc. Radiat. Transfer.

[36] Gordon I.E., Rothman L.S., Hargreaves R.J., Hashemi R., Karlovets E.V., Skinner F.M., Conway E.K., Hill C., Kochanov R.V., Tan Y., Wcisło P., Finenko A.A., Nelson K., Bernath P.F., Birk M., Boudon V., Campargue A., Chance K.V., Coustenis A., Drouin B.J., Flaud J.M., Gamache R.R., Hodges J.T., Jacquemart D., Mlawer E.J., Nikitin A.V., Perevalov V.I., Rotger M., Tennyson J., Toon G.C., Tran H., Tyuterev V.G., Adkins E.M., Baker A., Barbe A., Canè E., Császár A.G., Dudaryonok A., Egorov O., Fleisher A.J., Fleurbaey H., Foltynowicz A., Furtenbacher T., Harrison J.J., Hartmann J.M., Horneman V.M., Huang X., Karman T., Karns J., Kassi S., Kleiner I., Kofman V., Kwabia–Tchana F., Lavrentieva N.N., Lee T.J., Long D.A., Lukashevskaya A.A., Lyulin O.M., Makhnev V.Y., Matt W., Massie S.T., Melosso M., Mikhailenko S.N., Mondelain D., Müller H.S.P., Naumenko O.V., Perrin A., Polyansky O.L., Raddaoui E., Raston P.L., Reed Z.D., Rey M., Richard C., Tóbiás R., Sadiek I., Schwenke D.W., Starikova E., Sung K., Tamassia F., Tashkun S.A., Vander Auwera J., Vasilenko I.A., Vigasin A.A., Villanueva G.L., Vispoel B., Wagner G., Yachmenev A., Yurchenko S.N. (2022). The HITRAN2020 molecular spectroscopic database. J. Quant. Spectrosc. Radiat. Transf..

[37] Delahaye T., Armante R., Scott N.A., Jacquinet-Husson N., Chédin A., Crépeau L., Crevoisier C., Douet V., Perrin A., Barbe A., Boudon V., Campargue A., Coudert L.H., Ebert V., Flaud J.M., Gamache R.R., Jacquemart D., Jolly A., Kwabia Tchana F., Kyuberis A., Li G., Lyulin O.M., Manceron L., Mikhailenko S., Moazzen-Ahmadi N., Müller H.S.P., Naumenko O.V., Nikitin A., Perevalov V.I., Richard C., Starikova E., Tashkun S.A., Tyuterev V.G., Vander Auwera J., Vispoel B., Yachmenev A., Yurchenko S. (2021). The 2020 edition of the GEISA spectroscopic database. J. Mol. Spectrosc..

[38] Perrin A., Manceron L., Flaud J.M., Kwabia-Tchana F., Armante R., Roy P., Doizi D. (2021). The new nitrogen dioxide (NO_2_) linelist in the GEISA database and first identification of the ν_1_+2ν_3_-ν_3_ band of ^14^N^16^O_2_. J. Mol. Spectrosc..

[39] Naumenko O.V., Lukashevskaya A.A., Kassi S., Béguier S., Campargue A. (2019). The ν_1_ + 3ν_3_ absorption band of nitrogen dioxide (^14^N^16^O_2_) by CRDS near 6000 cm^-1^. J. Quant. Spectrosc. Radiat. Transf..

[40] Volkers E.A., Bulthuis J., Stolte S., Jost R., Linnartz H. (2006). High resolution electronic study of ^16^O^14^N^16^O, ^16^O^14^N^18^O and ^18^O^14^N^18^O: a rovibronic survey covering 11800–14380 cm^-1^. J. Mol. Spectrosc..

[41] Brand J.C.D., Hardwick J.L., Teo K.E. (1976). Laser-excited fluorescence of ^15^NO_2_ and N^18^O_2_. Can. J. Phys..

[42] Hardwick J.L., Brand J.C.D. (1976). Anharmonic potential constants and the large amplitude bending vibration in nitrogen dioxide. Can. J. Phys..

[43] Marinina A.A., Jacquemart D., Krim L., Soulard P., Perevalov V.I. (2022). The ν_3_ band of ^16^O^14^N^18^O: line positions and intensities. J. Quant. Spectrosc. Radiat. Transfer.

[44] Orphal J., Ruth A.A. (2008). High-resolution Fourier-transform cavity-enhanced absorption spectroscopy in the near-infrared using an incoherent broad-band light source. Opt. Express.

[45] Ruth A.A., Orphal J., Fiedler S.E. (2007). Fourier-transform cavity-enhanced absorption spectroscopy using an incoherent broadband light source. Appl. Opt..

[46] O'Leary D.M., Ruth A.A., Dixneuf S., Orphal J., Varma R. (2012). The near infrared cavity-enhanced absorption spectrum of methyl cyanide. J. Quant. Spectrosc. Radiat. Transf..

[47] Chandran S., Varma R. (2016). Near infrared cavity enhanced absorption spectra of atmospherically relevant ether-1,4-Dioxane, Spectrochim. Acta A Mol. Biomol. Spectrosc..

[48] Raghunandan R., Orphal J., Ruth A.A. (2020). New bands of deuterated nitrous acid (DONO) in the near-infrared using FT-IBBCEAS. Chem. Phys. Lett..

[49] Fiedler S.E., Hese A., Ruth A.A. (2003). Chem. Phys. Lett..

[50] Bird G.R., Baird J.C., Jache A.W., Hodgeson J.A., Curl R., Kunkle A.C., Bransford J.W., Rastrup‐Andersen J., Rosenthal J. (1964). Microwave spectrum of NO_2_: fine structure and magnetic coupling. J. Chem. Phys..

