# Supplementary material for: Experimental observation of the ν1+3ν3 combination bands of 16O14N18O and 18O14N18O in the near infrared spectral region
Source: Heliyon. 2024 Jan 24;10(3):e24853. doi: 10.1016/j.heliyon.2024.e24853 (PMC10844122; doi:10.1016/j.heliyon.2024.e24853)
Supplement: Multimedia component 1 [file mmc1.pdf]

# Ro-vibrational line list of the $\nu_1+3\nu_3$ combination band of $^{16}O^{14}N^{18}O$

A list of 314 line position and corresponding absorption coefficients above $0.17\times10^{-6}$ $cm^{-1}$ is shown in Table S1 for the spectral range from 5870 to 5940 $cm^{-1}$. Lines overlapping with absorption lines of water were left out of this table.

**Table S1**: Line positions for the vibrational transition (103) ← (000) of $^{16}O^{14}N^{18}O$ measured by FT-IBBCEAS. Column 2 contains the experimentally determined ro-vibrational line positions in wavenumbers ($\tilde{\nu}_{exp}$), column 3 shows the measured absorption coefficients ($\alpha$).

| **$^{16}O^{14}N^{18}O$** # | **Experimental line position ($\tilde{\nu}_{exp}$)** [$cm^{-1}$] | **Experimental absorption coefficients ($\alpha$)** $10^{-6}$ [$cm^{-1}$] |
|---|---|---|
| 1 | 5870.364 | 0.52 |
| 2 | 5870.628 | 0.23 |
| 3 | 5870.899 | 0.28 |
| 4 | 5871.238 | 0.52 |
| 5 | 5871.434 | 0.27 |
| 6 | 5871.517 | 0.27 |
| 7 | 5871.607 | 0.18 |
| 8 | 5871.886 | 0.35 |
| 9 | 5871.961 | 0.20 |
| 10 | 5872.044 | 0.37 |
| 11 | 5872.391 | 0.19 |
| 12 | 5872.458 | 0.28 |
| 13 | 5872.654 | 0.37 |
| 14 | 5873.016 | 0.31 |
| 15 | 5873.212 | 0.30 |
| 16 | 5873.656 | 0.24 |
| 17 | 5873.784 | 0.17 |
| 18 | 5873.905 | 0.19 |
| 19 | 5874.131 | 0.34 |
| 20 | 5874.349 | 0.45 |
| 21 | 5874.470 | 0.32 |
| 22 | 5874.545 | 0.32 |
| 23 | 5874.658 | 0.18 |
| 24 | 5874.846 | 0.69 |
| 25 | 5874.929 | 0.55 |
| 26 | 5875.426 | 0.18 |
| 27 | 5875.592 | 0.42 |
| 28 | 5876.029 | 0.49 |
| 29 | 5876.232 | 0.53 |
| 30 | 5876.413 | 0.39 |
| 31 | 5876.534 | 0.34 |
| 32 | 5876.715 | 0.29 |
| 33 | 5876.993 | 0.48 |
| 34 | 5877.189 | 0.27 |
| 35 | 5877.603 | 0.64 |

| | | |
|---|---|---|
| 36 | 5877.920 | 0.56 |
| 37 | 5878.153 | 0.24 |
| 38 | 5878.266 | 0.28 |
| 39 | 5878.432 | 0.35 |
| 40 | 5878.929 | 0.43 |
| 41 | 5879.389 | 1.18 |
| 42 | 5879.826 | 0.44 |
| 43 | 5880.255 | 0.40 |
| 44 | 5880.511 | 0.34 |
| 45 | 5880.624 | 0.92 |
| 46 | 5880.813 | 0.37 |
| 47 | 5880.978 | 0.25 |
| 48 | 5881.076 | 0.18 |
| 49 | 5881.204 | 0.47 |
| 50 | 5881.310 | 0.57 |
| 51 | 5881.558 | 0.65 |
| 52 | 5881.814 | 0.54 |
| 53 | 5882.116 | 0.78 |
| 54 | 5882.229 | 0.46 |
| 55 | 5882.327 | 0.22 |
| 56 | 5882.447 | 0.31 |
| 57 | 5882.545 | 0.39 |
| 58 | 5882.651 | 0.34 |
| 59 | 5882.816 | 0.55 |
| 60 | 5882.959 | 0.49 |
| 61 | 5883.577 | 0.87 |
| 62 | 5883.909 | 0.62 |
| 63 | 5884.089 | 0.56 |
| 64 | 5884.285 | 0.26 |
| 65 | 5884.481 | 0.19 |
| 66 | 5884.707 | 0.25 |
| 67 | 5885.197 | 0.63 |
| 68 | 5885.310 | 0.69 |
| 69 | 5885.702 | 0.39 |
| 70 | 5885.799 | 0.22 |
| 71 | 5885.980 | 0.23 |
| 72 | 5886.372 | 1.50 |
| 73 | 5886.786 | 0.43 |
| 74 | 5886.899 | 0.19 |
| 75 | 5887.735 | 2.19 |
| 76 | 5888.180 | 0.42 |
| 77 | 5888.564 | 0.87 |
| 78 | 5888.911 | 0.74 |
| 79 | 5889.016 | 1.10 |
| 80 | 5889.114 | 0.61 |
| 81 | 5889.235 | 0.39 |
| 82 | 5889.400 | 0.18 |
| 83 | 5889.762 | 0.26 |
| 84 | 5889.965 | 0.47 |

| | | |
|---|---|---|
| 85 | 5890.063 | 0.72 |
| 86 | 5890.274 | 0.83 |
| 87 | 5890.440 | 0.81 |
| 88 | 5890.628 | 0.45 |
| 89 | 5890.922 | 0.53 |
| 90 | 5891.314 | 0.38 |
| 91 | 5891.479 | 0.87 |
| 92 | 5891.607 | 0.77 |
| 93 | 5891.939 | 0.52 |
| 94 | 5892.662 | 0.90 |
| 95 | 5892.858 | 0.70 |
| 96 | 5893.061 | 1.02 |
| 97 | 5893.265 | 0.51 |
| 98 | 5893.845 | 0.86 |
| 99 | 5894.003 | 0.53 |
| 100 | 5894.086 | 0.74 |
| 101 | 5894.342 | 0.97 |
| 102 | 5894.493 | 0.55 |
| 103 | 5894.591 | 0.67 |
| 104 | 5894.907 | 0.60 |
| 105 | 5895.012 | 0.60 |
| 106 | 5895.314 | 0.89 |
| 107 | 5895.592 | 1.35 |
| 108 | 5895.849 | 0.46 |
| 109 | 5896.165 | 0.91 |
| 110 | 5896.519 | 0.73 |
| 111 | 5896.594 | 0.73 |
| 112 | 5896.715 | 0.32 |
| 113 | 5896.850 | 1.04 |
| 114 | 5896.971 | 0.17 |
| 115 | 5897.257 | 0.79 |
| 116 | 5897.626 | 0.55 |
| 117 | 5897.694 | 0.68 |
| 118 | 5898.063 | 1.25 |
| 119 | 5898.365 | 1.12 |
| 120 | 5898.493 | 0.17 |
| 121 | 5898.628 | 0.70 |
| 122 | 5898.847 | 0.63 |
| 123 | 5898.982 | 0.22 |
| 124 | 5899.088 | 0.69 |
| 125 | 5899.276 | 0.89 |
| 126 | 5899.359 | 0.17 |
| 127 | 5899.593 | 0.64 |
| 128 | 5899.721 | 0.39 |
| 129 | 5899.909 | 0.62 |
| 130 | 5900.007 | 0.74 |
| 131 | 5900.195 | 0.18 |
| 132 | 5900.308 | 0.63 |
| 133 | 5900.466 | 1.31 |

| | | |
|---|---|---|
| 134 | 5900.805 | 0.71 |
| 135 | 5901.385 | 0.20 |
| 136 | 5901.506 | 1.23 |
| 137 | 5901.988 | 0.61 |
| 138 | 5902.229 | 0.79 |
| 139 | 5902.726 | 1.22 |
| 140 | 5903.080 | 0.26 |
| 141 | 5903.163 | 0.49 |
| 142 | 5903.284 | 0.75 |
| 143 | 5903.427 | 0.52 |
| 144 | 5903.547 | 0.56 |
| 145 | 5903.653 | 0.18 |
| 146 | 5903.751 | 0.19 |
| 147 | 5903.864 | 1.03 |
| 148 | 5904.060 | 0.20 |
| 149 | 5904.331 | 1.03 |
| 150 | 5904.745 | 0.26 |
| 151 | 5905.001 | 1.06 |
| 152 | 5905.099 | 0.32 |
| 153 | 5905.393 | 1.30 |
| 154 | 5905.664 | 0.82 |
| 155 | 5905.935 | 0.40 |
| 156 | 5906.056 | 0.82 |
| 157 | 5906.402 | 1.65 |
| 158 | 5906.538 | 0.61 |
| 159 | 5906.772 | 0.89 |
| 160 | 5907.020 | 0.60 |
| 161 | 5907.126 | 1.53 |
| 162 | 5907.224 | 1.11 |
| 163 | 5907.397 | 0.65 |
| 164 | 5907.517 | 0.34 |
| 165 | 5907.608 | 0.56 |
| 166 | 5908.165 | 1.18 |
| 167 | 5908.286 | 0.52 |
| 168 | 5908.376 | 0.81 |
| 169 | 5908.482 | 0.29 |
| 170 | 5908.903 | 0.66 |
| 171 | 5909.032 | 0.36 |
| 172 | 5909.175 | 0.84 |
| 173 | 5909.725 | 0.85 |
| 174 | 5909.935 | 0.74 |
| 175 | 5910.056 | 0.68 |
| 176 | 5910.395 | 0.79 |
| 177 | 5910.591 | 0.45 |
| 178 | 5910.749 | 0.43 |
| 179 | 5910.937 | 0.47 |
| 180 | 5911.163 | 1.43 |
| 181 | 5911.299 | 0.32 |
| 182 | 5911.412 | 0.44 |

| | | |
|---|---|---|
| 183 | 5911.548 | 0.50 |
| 184 | 5911.962 | 0.73 |
| 185 | 5912.090 | 1.64 |
| 186 | 5912.421 | 0.59 |
| 187 | 5912.738 | 0.51 |
| 188 | 5912.934 | 0.70 |
| 189 | 5913.047 | 0.79 |
| 190 | 5913.212 | 0.34 |
| 191 | 5913.363 | 0.73 |
| 192 | 5913.566 | 0.20 |
| 193 | 5913.853 | 0.73 |
| 194 | 5913.958 | 0.71 |
| 195 | 5914.071 | 0.46 |
| 196 | 5914.350 | 0.65 |
| 197 | 5914.433 | 0.80 |
| 198 | 5914.666 | 0.48 |
| 199 | 5914.764 | 0.71 |
| 200 | 5914.885 | 0.68 |
| 201 | 5915.570 | 1.02 |
| 202 | 5915.713 | 0.97 |
| 203 | 5915.872 | 0.56 |
| 204 | 5916.060 | 1.61 |
| 205 | 5916.195 | 0.83 |
| 206 | 5916.497 | 0.81 |
| 207 | 5916.587 | 1.15 |
| 208 | 5916.738 | 0.63 |
| 209 | 5916.873 | 0.55 |
| 210 | 5917.084 | 1.15 |
| 211 | 5917.167 | 0.69 |
| 212 | 5917.386 | 0.95 |
| 213 | 5917.499 | 0.98 |
| 214 | 5918.305 | 0.60 |
| 215 | 5918.425 | 0.63 |
| 216 | 5918.651 | 0.63 |
| 217 | 5918.749 | 0.67 |
| 218 | 5918.847 | 1.09 |
| 219 | 5919.126 | 0.31 |
| 220 | 5919.239 | 0.85 |
| 221 | 5919.623 | 0.87 |
| 222 | 5919.954 | 0.86 |
| 223 | 5920.316 | 0.66 |
| 224 | 5920.437 | 0.85 |
| 225 | 5920.640 | 0.89 |
| 226 | 5920.941 | 1.05 |
| 227 | 5921.288 | 0.88 |
| 228 | 5921.476 | 1.25 |
| 229 | 5921.695 | 0.55 |
| 230 | 5921.988 | 1.36 |
| 231 | 5922.101 | 0.81 |

| | | |
|---|---|---|
| 232 | 5922.282 | 0.72 |
| 233 | 5922.463 | 1.21 |
| 234 | 5922.568 | 1.18 |
| 235 | 5922.779 | 0.94 |
| 236 | 5922.930 | 2.08 |
| 237 | 5923.156 | 0.90 |
| 238 | 5923.375 | 1.28 |
| 239 | 5923.548 | 0.50 |
| 240 | 5923.751 | 1.73 |
| 241 | 5923.887 | 0.24 |
| 242 | 5924.459 | 0.54 |
| 243 | 5924.557 | 0.89 |
| 244 | 5924.715 | 0.95 |
| 245 | 5924.821 | 1.05 |
| 246 | 5924.926 | 1.27 |
| 247 | 5925.250 | 1.01 |
| 248 | 5925.333 | 1.58 |
| 249 | 5925.642 | 1.09 |
| 250 | 5925.845 | 2.31 |
| 251 | 5926.139 | 0.80 |
| 252 | 5926.290 | 2.45 |
| 253 | 5926.651 | 0.93 |
| 254 | 5926.757 | 1.31 |
| 255 | 5926.877 | 1.36 |
| 256 | 5927.066 | 2.59 |
| 257 | 5927.209 | 1.53 |
| 258 | 5927.578 | 3.20 |
| 259 | 5927.721 | 0.98 |
| 260 | 5927.932 | 1.64 |
| 261 | 5928.007 | 1.89 |
| 262 | 5928.113 | 2.05 |
| 263 | 5928.384 | 1.07 |
| 264 | 5928.580 | 2.04 |
| 265 | 5928.731 | 1.42 |
| 266 | 5928.859 | 0.71 |
| 267 | 5929.077 | 2.92 |
| 268 | 5929.250 | 0.63 |
| 269 | 5929.393 | 1.55 |
| 270 | 5929.574 | 1.41 |
| 271 | 5929.951 | 2.58 |
| 272 | 5930.230 | 1.29 |
| 273 | 5930.335 | 1.04 |
| 274 | 5930.456 | 2.54 |
| 275 | 5930.674 | 1.26 |
| 276 | 5930.742 | 1.66 |
| 277 | 5930.862 | 1.63 |
| 278 | 5931.058 | 1.22 |
| 279 | 5931.209 | 1.80 |
| 280 | 5931.367 | 2.01 |

| | | |
|---|---|---|
| 281 | 5931.653 | 1.82 |
| 282 | 5931.766 | 2.38 |
| 283 | 5931.955 | 0.90 |
| 284 | 5932.090 | 1.85 |
| 285 | 5932.467 | 1.86 |
| 286 | 5932.610 | 1.53 |
| 287 | 5932.768 | 0.94 |
| 288 | 5932.874 | 1.59 |
| 289 | 5933.077 | 1.50 |
| 290 | 5933.198 | 1.08 |
| 291 | 5933.273 | 0.95 |
| 292 | 5933.394 | 1.57 |
| 293 | 5933.537 | 1.25 |
| 294 | 5933.635 | 1.36 |
| 295 | 5933.755 | 1.37 |
| 296 | 5933.861 | 2.43 |
| 297 | 5934.087 | 3.31 |
| 298 | 5934.275 | 0.97 |
| 299 | 5934.380 | 2.16 |
| 300 | 5934.644 | 2.08 |
| 301 | 5934.870 | 2.72 |
| 302 | 5935.013 | 1.97 |
| 303 | 5935.096 | 2.00 |
| 304 | 5935.217 | 0.85 |
| 305 | 5935.360 | 1.43 |
| 306 | 5935.548 | 1.34 |
| 307 | 5935.714 | 2.00 |
| 308 | 5935.849 | 0.78 |
| 309 | 5935.977 | 0.68 |
| 310 | 5936.113 | 1.03 |
| 311 | 5936.241 | 1.09 |
| 312 | 5936.550 | 0.29 |
| 313 | 5936.640 | 0.41 |
| 314 | 5936.768 | 0.54 |
